# Surface wave manipulation by plasmonic metasurface based on mode resonance

**DOI:** 10.1038/s41598-021-82948-0

**Published:** 2021-02-08

**Authors:** Baoshan Guo

**Affiliations:** Laser Micro/Nano Fabrication Laboratory, School of Mechanical Engineering, Beijing Institute of Technology, Beijing, 100081 China

**Keywords:** Integrated optics, Metamaterials

## Abstract

We proposed a method to manipulate the surface waves with a deep subwavelength metasurface by applying resonators with interfering mode resonance. The simulation results demonstrate that a single deep subwavelength obstructed groove can effectively control the propagation of surface terahertz (THz) waves by a small step increase (< 1/20 λ) of the depth or a slight change of refractive index (Δn = 0.1). The surface waves transmitted and reflected by the single groove can be controlled periodically by increasing the groove depth or refractive index with a high efficiency owing to the mode resonance between surface spoof plasmonics modes and groove cavity modes. The generated circle resonance mode provides a new idea for the development of THz devices. Importantly, the transmitted or reflected intensity of the surface wave is also enhanced by the Mode resonance. It is a simple and effective method to operate surface THz waves and manufacture more compact integrated optical devices in deep subwavelength scale.

## Introduction

The most important characteristic of plasmonics is to confine the electromagnetic (EM) energy and enhance the interaction between light and matter in the subwavelength range^1,2^, which has been applied in a wide range of fields, such as the miniaturization of photonic circuits^3,4^, near-field optics and microscopy^5,6^, biological sensors^7,8^, and photovoltaics^9,10^. At terahertz (THz) frequencies, various subwavelength metal structures based on spoof SPPs (SSPPs)^11–13^ have been developed to efficiently control the properties of THz fields^14–20^. Based on this, a series of new THz devices with high performance have been developed and applied in different areas, such as THz sensors^21–23^, THz spectrum and imaging detection^24–26^, and THz communication^27^. One of the most representative devices is the one-dimensional THz metal grating waveguide, which has been designed and fabricated into different groove shapes, including rectangular, inclined rectangular, trapezoidal, V-shaped, serrated and meniscus grooves^28–37^. If the surface grating has gradient depth^38–42^, gradient period^43^, and vertical or downward pyramid grooves^44^, it can realize the so-called "trapped rainbow" that has been proved to be a reflection rather than a real EM wave trapping^45,46^. In addition to the groove shape of the grating, the surface EM wave can also be controlled by changing the local refractive index of the dielectric layer covering the metal grating, just as a new dielectric grating is formed above the metal grating^47^. In fact, the change of refractive index in a groove of the metal grating itself can also significantly change the transmission characteristics of the surface wave, which will be discussed below. Furthermore, detailed understanding of the physical mechanism underlying single-groove manipulation of EM waves still needs study whether there is a covered dielectric layer or not. In this paper, it is revealed that the controlling of surface EM waves can be achieved by simply changing the depth or refractive index of a single deep subwavelength groove in the metal grating, and detailed physical explanation is given. With the increase of the groove depth or refractive index, the surface wave propagation characteristics show obvious periodic changes, which is essentially caused by the mode resonance between the surface modes and the groove cavity modes, similar to the Fano resonance^47–50^. Moreover, under the mode resonance, the grating surface will form high-intensity local standing waves, which can be applied to deep subwavelength resonators or spasers.

## Simulation and discussion

**Figure 1:**
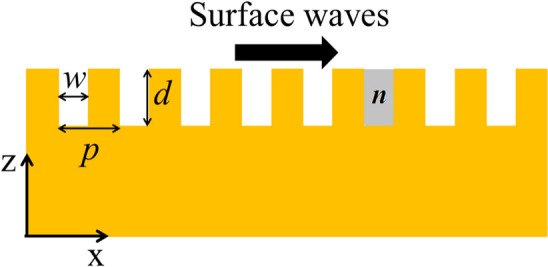
Schematic of metal grating showing width (*w*), period (*p*), depth (*d*), and refractive index (*n*) of the material filling in the single grey groove. The surface waves propagate along the direction of the X axis as shown by the black arrow.

The metal grating (Fig. 1) has rectangular grooves with depth (*d*), width (*w*), and period (*p*). By simply adjusting the main parameters of depth (*d*), width (*w*), and period (*p*), it can be designed to propagate or decelerate EM waves of different frequencies, because the dispersion property of the grating is determined by theses parameters^11,13,45^. In addition, as previously mentioned, the refractive index (*n*) of the material filling in the grooves can also affect the propagation of the EM waves. To further investigate how to control the surface EM waves by the mode resonance between the grating and its single groove, a metal grating model (Fig. 1) is established and simulated by the finite difference time domain (FDTD) method. A special groove with gradual changes in refractive index (*n*) is marked in grey in Fig. 1. The grating model with a uniform cell of ∆x = ∆z = 1 μm is treated as a perfect conductor (PEC) during the FDTD simulation, and surrounded by a perfectly matched absorption layer. The depth and refractive index of the filling media of the single grey groove are the main parameters to control the propagation of surface waves. At 1000 μm to the left of the single grey groove, a p-polarized (Hy, Ex, Ez) THz source is introduced in the form of end fire excitation and used to excite the surface THz waves to propagate from left to right as shown by the black arrow in Fig. 1.

**Figure 2:**
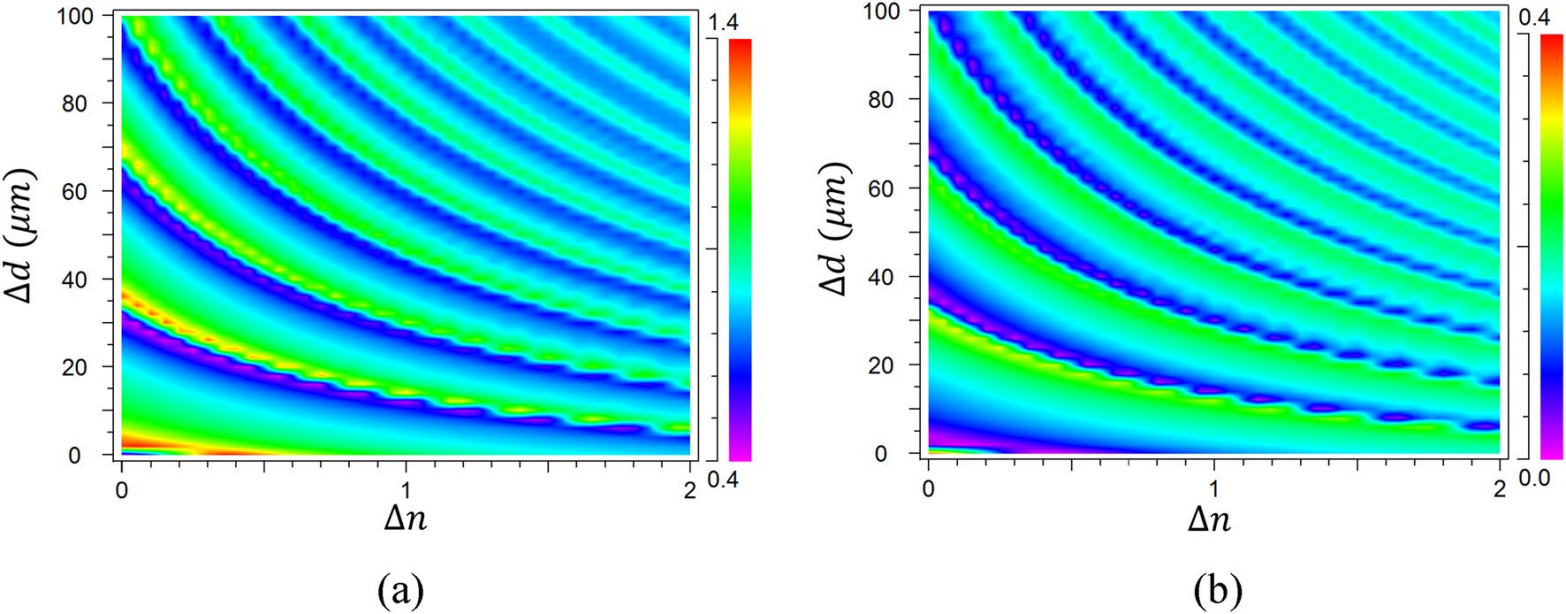
Intensity of surface waves at 3.75 THz before (**a**) and after (**b**) the single grey groove of varying depth (Δ*d*) and refractive index (Δ*n*).

**Figure 3:**
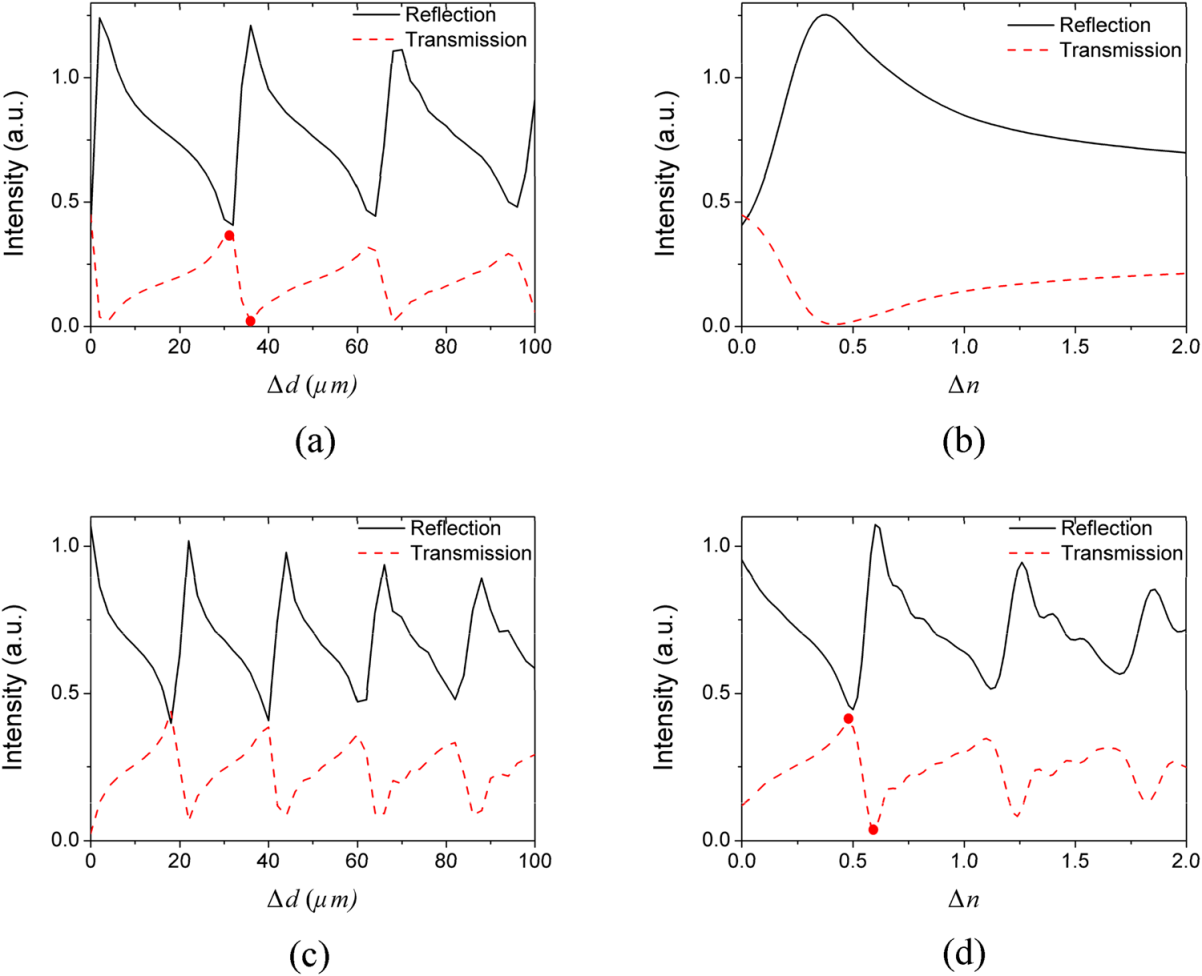
Intensity of surface waves before (black solid line) and after (red dashed line) the single grey groove of varying depth, Δ*n* = 0 (**a**), Δ*n* = 0.5 (**c**); and various refractive indices, Δ*d* = 0 μm (**b**), Δ*d* = 40 μm (**d**).

The cutoff frequency of a metal grating with period of 20 μm, width of 10 μm and depth of 10 μm is lower than 4.6 THz^45^. Hence, a source of 3.75 THz is used to excite the propagating surface wave, which propagates smoothly along the grating until it encounters the special grey groove (as shown in Fig. 1). In the simulation process, we only change the depth and refractive index of the single grey groove. The surface wave intensities before and after the single groove are recorded by two monitors, respectively, so as to analyze the surface wave intensities reflected and transmitted by the single groove. According to the FDTD simulation results (Fig. 2), the surface wave intensities before (reflection) and after (transmission) the single groove are all changed periodically with the increase of groove depth (Δ*d*) or refractive index (Δ*n*). The intensity of electric field at localized points could be larger than “1” because of the joint contribution of mode resonance between plasmonic surface mode and groove cavity mode, and the interference between reflection surface wave and incident wave. For a grating working as a normal waveguide, the monitored intensity values at the reflection and transmission points are almost same and far below “1” as shown in Fig. 3. For example, at the zero point of Fig. 3a, Δ*n* = 0, Δ*d* = 0 μm, the grey groove is exactly same with other grooves, and the grating becomes a normal grating. Therefore, the transmission and reflection intensity are all approximately equal to “0.4”, which is a base intensity of the surface wave. When the reflection gradually increases and the transmission gradually weakens, the measured reflection intensity starts to be greater than “0.4”, while the transmission intensity is gradually less than “0.4”. Hence, the reflection and transmission intensity value are very different. It is also the reason why the scale bar value in Fig. 2a, b are different. The reflection intensity defined here is changing on the base intensity of “0.4” as shown in Fig. 2a.

For the single groove of Δ*n* = 0 (*n* = 1) (Fig. 3a), the changing period of the surface wave intensity with the depth is approximately 30 μm. And the intensity peak shows a clear asymmetry shape, which is similar to the Fano resonance between the grating surface modes and groove cavity modes. When Δ*n* = 0.5 (*n* = 1.5) (Fig. 3c), the changing period with depth is decreased to 20 μm (equals to 30 μm / 1.5), which means the effective wavelength is decreased corresponding to the increase of refractive index. For Δ*d* = 0 μm (Fig. 3b), the reflection of the surface wave is sharply increased first with the increase of Δ*n*, and is decreased slowly after the peak (Δ*n ≈* 0.4) (black line in Fig. 3b). The transmission intensity (black line in Fig. 3b) of the single groove shows a trend opposite to the reflection intensity (red dashed line in Fig. 3b). However, there is no obvious periodicity because the single groove is not deep enough to form obvious mode resonance. When the depth of the single groove is increased to 50 μm (Δ*d* = 40 μm) (Fig. 3d), a serials of strong periodical mode resonance are generated with the increase of Δ*n*.

**Figure 4:**
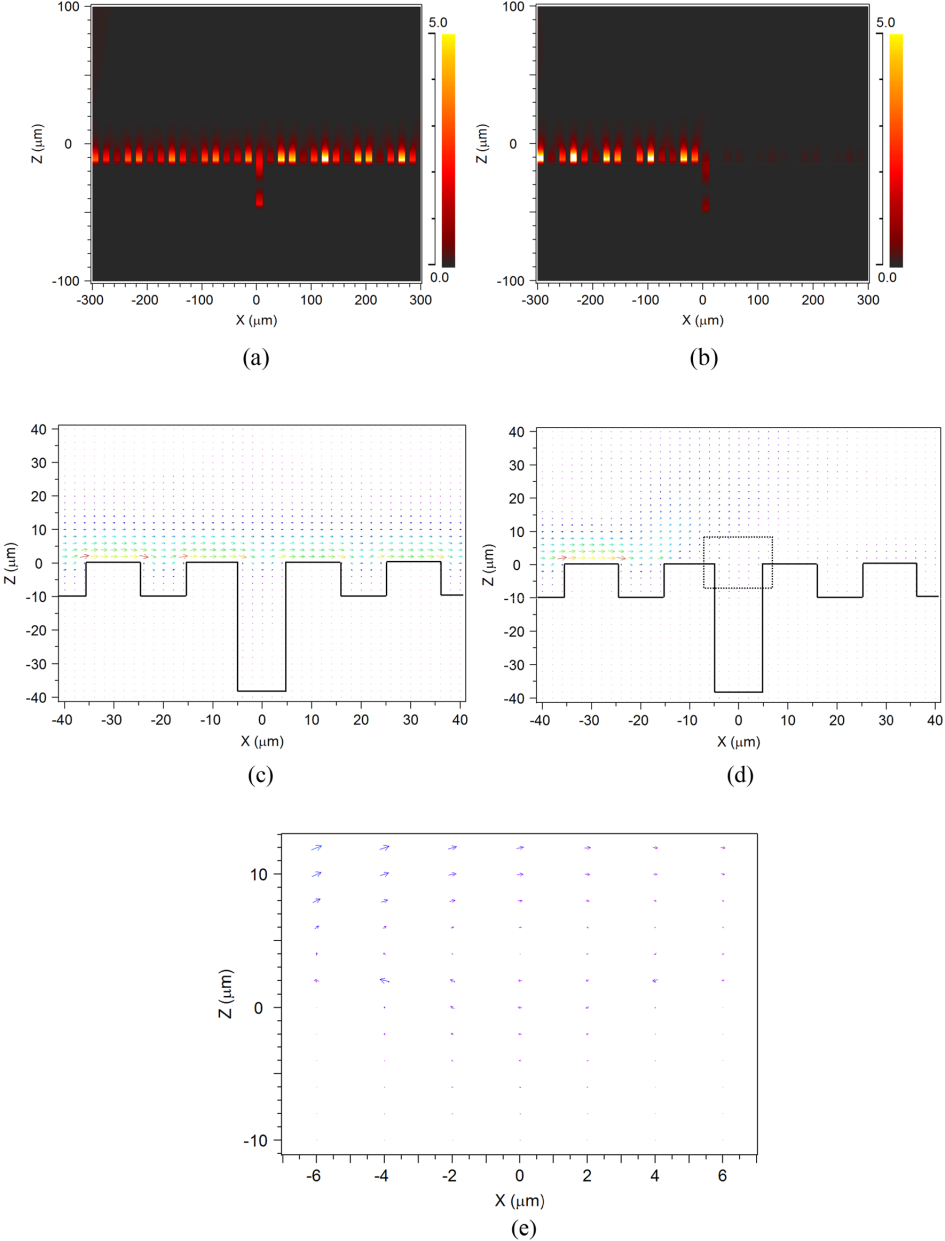
Two-dimensional field distribution of EM wave with a frequency of 3.75 THz obtained through grating with a single obstructed groove of varying depth (Δ*n* = 0). The increased depths of the obstructed groove are Δ*d* = 32 μm (**a**), Δ*d* = 36 μm (**b**). The Poynting vector distribution near the single obstructed groove with Δ*d* = 32 μm (**c**), Δ*d* = 36 μm (**d**). The Poynting vector distribution in the enlarged region marked by black dotted line in center of (**d**) is shown in (**e**).

Utilizing the sharp asymmetry property of the Fano resonance, we can control the surface waves with high sensitivity and efficiency. As an example, two points marked in Fig. 3a and two points marked in Fig. 3d are chosen, respectively, to demonstrate the ability of surface wave manipulation. The two points marked in Fig. 3a correspond to the transmission peak at Δ*d* = 32 μm and transmission valley at Δ*d* = 36 μm, respectively for the surface wave (Δ*n* = 0). It means that the excited surface wave with frequency of 3.75 THz can be propagated along the surface grating and transmitted over the single groove (Fig. 4a) when the single groove depth is 42 μm (Δ*d* = 32 μm). However, when Δ*d* = 36 μm, the propagated surface wave is almost totally blocked (Fig. 4b). Hence, we can precisely control the transmission or reflection of the surface wave by adjusting the depth of the single grey groove within a step range of 4 μm (< 1/20 λ).

**Figure 5:**
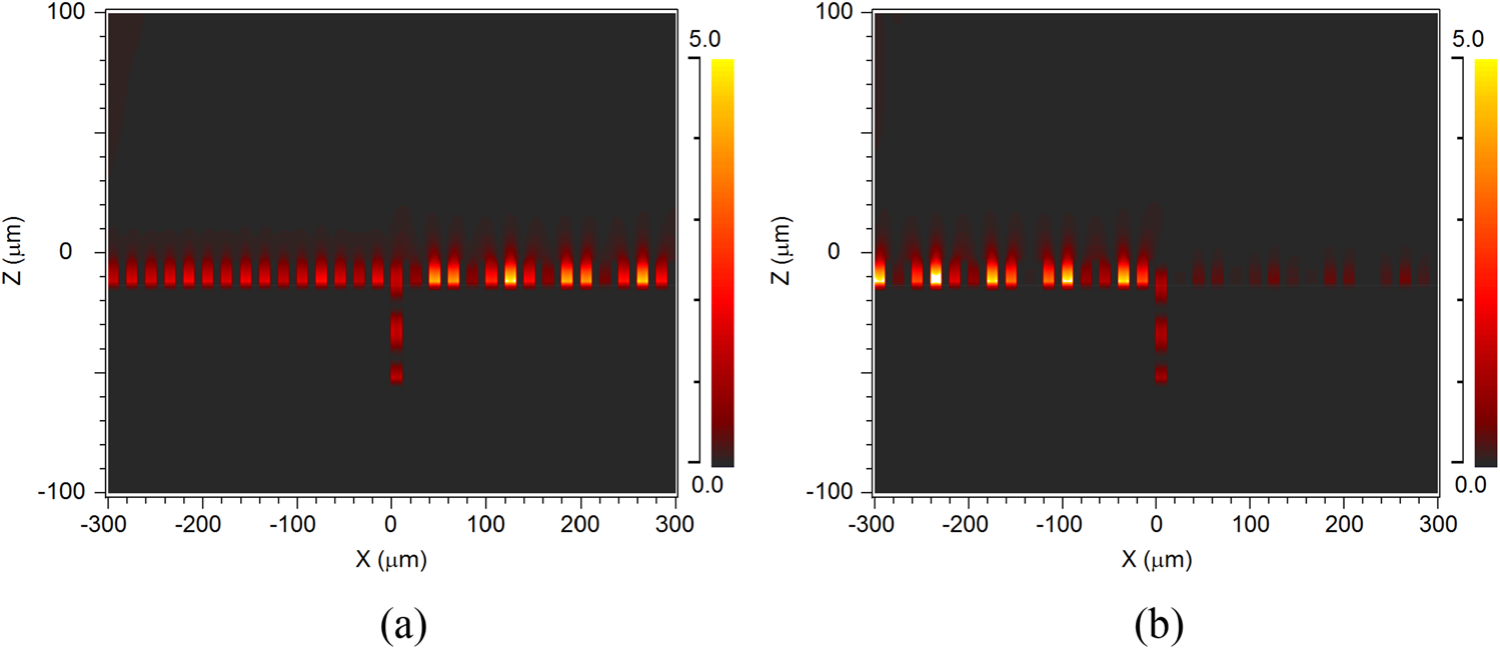
Two-dimensional field distribution of EM wave with a frequency of 3.75 THz obtained through grating with a single grey groove of varying refractive index (Δ*d* = 40 μm). The increased refractive indices of the single groove are Δ*n* = 0.5 (**a**), Δ*n* = 0.6 (**b**).

In order to further understanding the mode interaction, the Poynting vector distributions around the single obstructed groove with different depth are shown in Fig. 4c (Δ*d* = 32 μm), and Fig. 4d (Δ*d* = 36 μm), respectively. For Δ*d* = 32 μm, there are no resonance between the surface spoof plasmonics mode and groove cavity mode, and the metasurface works as a high efficient waveguide. The plasmonics mode is propagated along the surface and coupled from groove to groove (Fig. 4c). The groove cavity mode is formed in the single obstructed groove which is similar to a Fabry–Perot mode, which can be seen in Fig. 5 more clearly. However, when Δ*d* = 36 μm, the resonance between the surface spoof plasmonics mode and groove cavity mode forms a new circle resonance mode as shown in Fig. 4e (the enlarged region marked by black dotted line in center of Fig. 4d), which is the physical reason of the deep subwavelength single grey groove can manipulate the surface wave.

**Figure 6:**
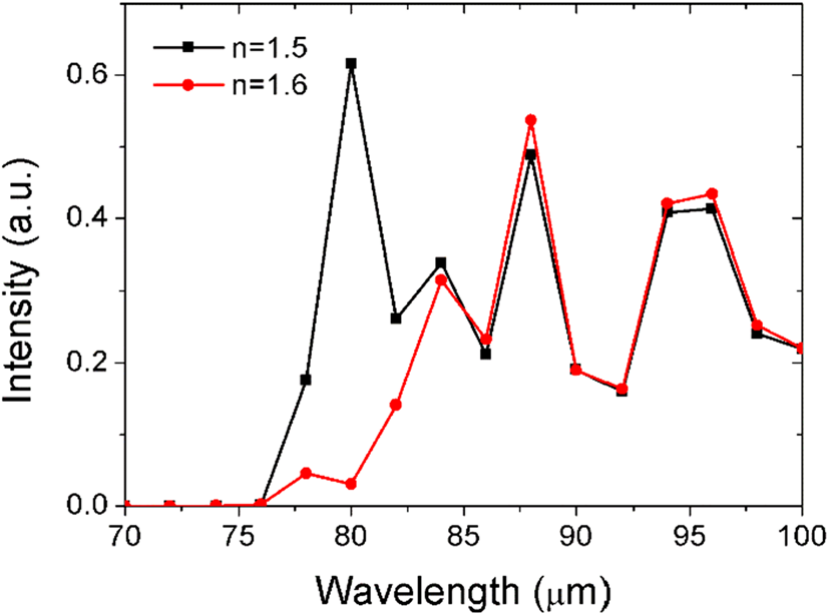
The transmission spectrum of the single groove (Δ*d* = 40 μm) with different refractive index: n = 1.5 (square dotted black line), n = 1.6 (circle dotted red line).

Similarly, the two points marked in Fig. 3d correspond to the transmission peak at Δ*n* = 0.5 and transmission valley at Δ*n* = 0.6, respectively (Δ*d* = 40 μm). Therefore, the propagated surface wave with frequency of 3.75 THz can be transmitted when Δ*n* = 0.5, but blocked when Δ*n* = 0.6 (Fig. 5). It means that we can also control the transmission or reflection of the surface wave with a slight changing of refractive index (Δ*n* = 0.1). Importantly, the transmitted or reflected waves are all enhanced to higher intensity than the normal surface waves by the mode resonance, which provide a new and easy way to obtain the controllable surface waves with enhanced intensity. If we want to use it for the refractive index sensing, the sensing sensitivity (*Δλ/Δn*) can be estimated by the transmission curves at different refractive index as shown in Fig. 6 in the manuscript. Here, the transmission peak is not shifted with the refractive index changing as traditional sensor. The main difference of the square dotted black line (*n* = 1.5) and circle dotted red line (*n* = 1.6) is the existence of the transmission peak from wavelength of 78 μm to 82 μm. Hence, *Δλ* here could be the peak width (~ 4 μm). The refractive index sensing sensitivity (*Δλ/Δn*) is approximately 40 μm/RIU, which is already a competitive value comparing to typical sensors^22,51–53^.

**Figure 7:**
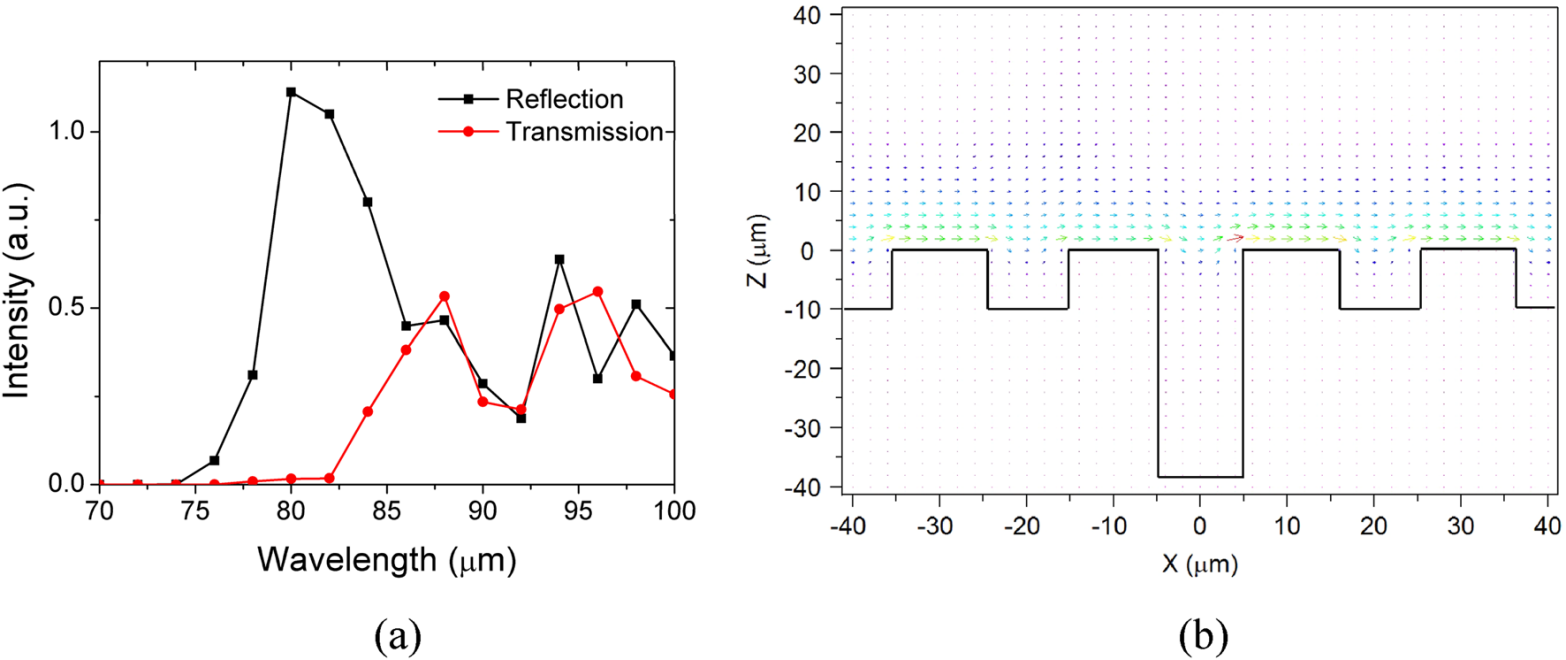
The reflection (square dotted black line) and transmission spectrum (circle dotted red line) of the single obstructed groove (Δ*n* = 0, Δ*d* = 36 μm) at around 3.75 THz (corresponding to wavelength of 80 μm) (**a**), The Poynting vector distribution of the metasurface at wavelength of 84 μm (**b**).

At last, the transmission spectrum of the single obstructed groove (Δ*n* = 0, Δ*d* = 36 μm) at around 3.75 THz (corresponding to wavelength of 80 μm) is presented in Fig. 7a. The plasmonic surface modes begin to appear from wavelength of 75 μm (black reflection curve), and all blocked by the mode resonance effect. The resonance effect begins to disappear at wavelength of 82 μm (red transmission curve), which demonstrates that the metasurface has a high sensitivity spectral response. The full-wave at half maximum (FWHM) of the square dotted black line peak is approximately 6 μm, which could be considered as the FWHM of the mode resonance spectrum peak. After wavelength of 85 μm, the reflection intensity and transmission intensity go into the same level. It means that the reflection field faded, and the metasurface works as a waveguide again. The Poynting vector distribution at wavelength of 84 μm is shown in Fig. 7b. The circle resonance mode shown in Fig. 4e is no longer exist, which also verifies the high sensitivity spectral response of the mode resonance.

## Conclusion

It has been demonstrated that the propagation characteristics of surface waves can be efficiently controlled by a single deep subwavelength groove with only 4 μm depth changing (< 1/20 λ) or refractive index changing of 0.1. We can use a few or more special grooves of different depth or refractive index located at different positions to manipulate the EM waves of different frequencies by the mode resonance between the plasmonic mode and groove cavity mode. The physical reason is the new circle resonance mode generation. It provides a new idea for the development of new THz devices. In addition, the intensity of the transmitted or reflected waves are all enhanced by the mode resonance, which is important for high sensitivity detection. From the application point of view, the depth or refractive index of the single groove is easy to change, which can provide a simple scheme to efficiently control the transmission characteristics of the EM wave at the deep subwavelength scale. Hence, it can further promote the development of THz compact devices and integrated technology.
